# Cerebrovascular autoregulation in hypoxia: quantitative insights from arterial spin labeling

**DOI:** 10.3389/fnins.2025.1672726

**Published:** 2025-09-16

**Authors:** Abir Troudi Habibi, Julia Micaux, Franck Mauconduit, Marion Noulhiane

**Affiliations:** ^1^Joliot Institute, University of Paris-Saclay, CEA, NeuroSpin, UNIACT, Gif-sur-Yvette, France; ^2^University of Paris Cite, Inserm, U1141 NeuroDiderot, InDev Team, Paris, France; ^3^CEA, NeuroSpin, CNRS, University of Paris-Saclay, Gif-sur-Yvette, France

**Keywords:** perfusion MRI, ASL, CBF, hypoxia, cerebrovascular autoregulation

## Abstract

Hypoxia, defined as an insufficient oxygen supply relative to metabolic demand, induces a cascade of cerebrovascular and metabolic responses aimed at preserving cerebral homeostasis. These responses vary depending on the temporal profile of exposure, with acute (e.g., hypoxic–ischemic encephalopathy, acute high-altitude exposure) versus chronic (e.g., obstructive sleep apnea, long-term altitude residence) conditions, and may range from compensatory vasodilation to long-term maladaptive remodeling. Arterial Spin Labeling (ASL) MRI offers a quantitative, non-invasive, and contrast-free method to assess cerebral perfusion, making it well-suited to characterize the spatial and temporal dynamics of these responses. This narrative review critically examines the application of ASL to quantify key hemodynamic parameters, including cerebral blood flow (CBF), arterial transit time (ATT), cerebrovascular reactivity (CVR), and, when integrated with complementary models, cerebral metabolic rate of oxygen consumption (CMRO₂), in the context of hypoxia. By synthesizing evidence from both environmental and pathological models induced by hypoxia, we highlight how ASL captures early signatures of cerebrovascular adaptation, impaired autoregulation, and emerging neurovascular dysfunction. Particular emphasis is placed on the potential of ASL-derived metrics to serve as early biomarkers for hypoxia-induced risk, enabling non-invasive longitudinal tracking of vascular integrity in both clinical and subclinical populations. Overall, ASL emerges as a powerful modality for elucidating the mechanisms of neurovascular adaptation to hypoxia and for supporting precision diagnostics in disorders where oxygen insufficiency constitutes a key pathophysiological driver.

## 1 Introduction

Hypoxia, defined as an insufficient supply of oxygen to meet tissue metabolic demands, represents a fundamental physiological challenge for the brain, the organ with the highest oxygen consumption per unit mass. While hypoxia can arise as a gradual, chronic exposure or as an acute, transient episode, both scenarios act as aggressive etiological stressors on a brain initially functioning within homeostatic limits. In response, the brain mobilizes a series of tightly regulated compensatory mechanisms, such as cerebrovascular autoregulation, vasodilation, and metabolic adaptation, to preserve oxygen delivery and maintain neural viability (Mallat et al., 2022).

Arterial Spin Labeling (ASL) MRI has emerged as a powerful neuroimaging technique for investigating cerebral perfusion in both physiological and pathological states of hypoxia. By magnetically labeling arterial blood water as an endogenous tracer, ASL provides a non-invasive, contrast-free, and quantitative assessment of cerebral blood flow (CBF) in absolute units (mL/100 g/min), with high regional specificity (Wang et al., 2002; Taso and Alsop, 2024; Wong and Maller, 2016). Importantly, beyond static measures of flow, ASL allows estimation of arterial transit time (ATT) and cerebrovascular reactivity (CVR), parameters that reflect vascular efficiency, reserve capacity, and autoregulatory integrity. These derived metrics enrich our understanding of the brain’s dynamic vascular responses to hypoxia, including changes in the cerebral metabolic rate of oxygen (CMRO₂), which further illuminate the interplay between supply and demand under oxygen-limited conditions.

Originally developed for neurovascular and oncological applications such as neurodegeneration and tumor imaging (Noguchi et al., 2008; Troudi et al., 2023; Troudi et al., 2022), dementia (Grade et al., 2015), ASL has gained traction in conditions involving altered oxygen availability. In obstructive sleep apnea (OSA; Harris et al., 2013), chronic mountain sickness (Ainslie and Subudhi, 2014), and high-altitude adaptation, ASL studies consistently report perfusion changes in regions governing autonomic regulation, memory, and executive function. These findings support the idea that hypoxia induces region-specific cerebrovascular alterations that may precede overt structural damage. Furthermore, metrics such as prolonged ATT or reduced CVR have been linked to impaired vascular reserve and cognitive vulnerability in aging and small vessel disease (Dai et al., 2017; Neumann et al., 2022), underscoring clinically relevant biomarkers for hypoxia-induced neurovascular dysfunction.

Despite growing interest in ASL under hypoxic conditions, several key questions remain unresolved: How does cerebral autoregulation evolve during the transition from acute to chronic hypoxia? Can ASL detect early biomarkers of maladaptive vascular remodeling or incipient neurocognitive decline? What distinguishes *adaptive* from *pathological* perfusion responses at the regional level? most clinical imaging protocols still overlook subtle or dynamic cerebrovascular changes that occur in the absence of gross structural abnormalities, a gap that ASL, with its sensitivity to early hemodynamic shifts in structurally intact brain systems, may help bridge.

This narrative review synthesizes current evidence on the use of ASL to investigate the brain’s vascular responses to hypoxia, both environmental and pathological. We focus on:

- The physiological basis of cerebrovascular responses to hypoxia and their detectability via ASL;
- The differential impact of acute vs. chronic hypoxia on CBF and related perfusion metrics;
- The potential of ASL-derived parameters (CBF, ATT, CVR, and CMRO_2_) as biomarkers of cerebrovascular dysfunction;
- The translational implications of ASL in clinical diagnosis, risk stratification, and longitudinal monitoring in hypoxia-related brain disorders.

By consolidating findings across models, this review underscores ASL’s unique ability to capture both early compensatory responses and later-stage perfusion deficits, offering a window into neurovascular vulnerability and resilience before irreversible damage occurs.

## 2 An overview of ASL perfusion imaging

### 2.1 Principles of ASL

**Figure 1:**
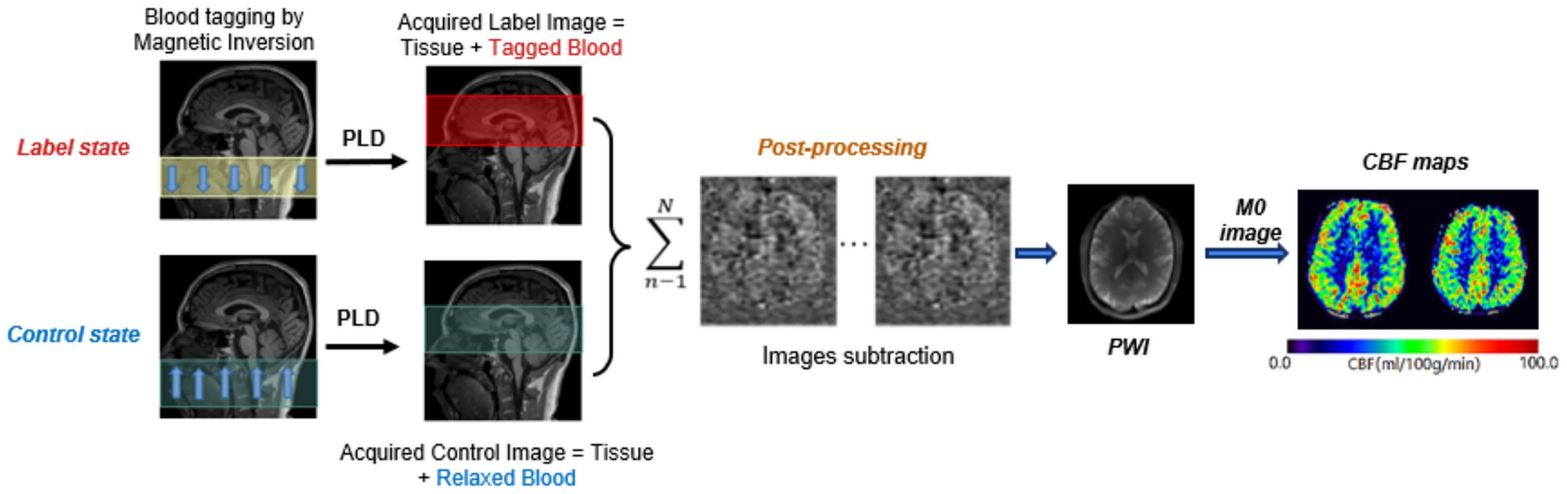
Illustration of the fundamental concept of ASL and subsequent image processing. PLD, post-labeling delay; PWI, perfusion weighted image; CBF, cerebral blood flow.

ASL is a non-invasive MRI technique that measures cerebral perfusion using magnetically labeled arterial blood water as an endogenous tracer (Kwong et al., 1995). Labeling is achieved by applying a radiofrequency (RF) pulse upstream from the imaging volume, which inverts the magnetization of inflowing arterial blood. After a post-labeling delay (PLD), the labeled blood reaches the brain tissue and exchanges with tissue water, generating a signal change proportional to CBF (Figure 1; Williams et al., 1992). The standard ASL acquisition alternates between two conditions: *Label image*: arterial blood is inverted, leading to reduced tissue signal. *Control image*: no inversion is applied, serving as a baseline. By subtracting the label from the control image, a perfusion-weighted image (PWI) is obtained, isolating the signal difference related to tissue perfusion (Noguchi et al., 2008; Suzuki et al., 2024). This subtraction method reduces static tissue contributions, enhancing sensitivity to flow-related changes.

**Figure 2:**
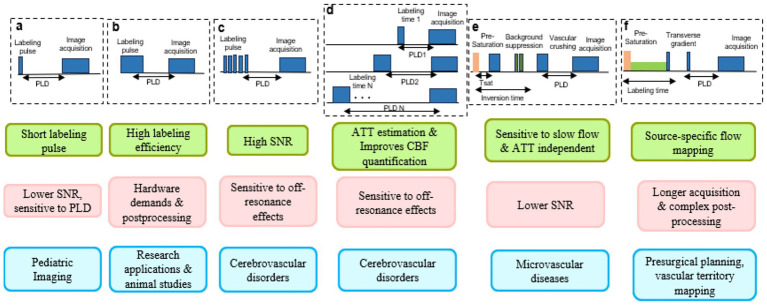
Arterial spin labeling techniques. PLD, post-labeling delay; **(a)** Pulsed ASL; **(b)** Continuous ASL; **(c)** Pseudo-Continuous ASL; **(d)** Multi-delay ASL; **(e)** Velocity selective ASL; **(f)** Vessel-encoded ASL. Green panels represent the Pros; Pink panels represent the Cons; blue panels represent the applications.

Several ASL techniques exist, differing in labeling strategy, sequence design, and sensitivity to physiological conditions (Figure 2; Table 1):

- Pulsed ASL (PASL) uses short RF pulses to label a large arterial region, offering a simple and rapid acquisition. However, it typically produces a lower SNR and may be more sensitive to arterial transit time effects compared to continuous or pseudo-continuous ASL techniques (Golay et al., 2004).
- Continuous ASL (CASL), introduced by Williams et al. (1992), applies a long RF pulse (2–4 s) for sustained labeling, yielding higher signal but requiring hardware capable of handling prolonged RF exposure (Detre et al., 1992; Williams et al., 1992; Detre and Alsop, 1999; Wang et al., 2002).
- Pseudo-continuous ASL (pCASL) mimics CASL using rapid short pulses, offering high efficiency with lower specific absorption rate, and is now the clinical standard (Alsop et al., 2015; Suzuki et al., 2024; Dai et al., 2008).
- Multi-delay ASL (MDASL) acquires multiple PLDs, enabling better modeling of transit delays and more accurate CBF quantification, though with increased acquisition time and complexity (Ishida, 2023; Woods et al., 2024).
- Velocity-selective ASL (VSASL) tags blood based on velocity rather than location, improving sensitivity to slow-flow regions and enhancing microvascular assessments (Qin et al., 2022).
- Vessel-encoded ASL (VE-ASL) targets specific arteries to map territorial perfusion, valuable for studying vascular territories in stroke or malformations (Wong, 2007).

**Table 1:** Comparative summary of ASL techniques: advantages, limitations, and common applications.

Labeling technique	Advantages	Limitations	Applications
PASL	Short labeling pulse, no RF power deposition issues	Lower SNR, sensitive to PLD	Pediatric Imaging
CASL	High labeling efficiency	High RF power deposition, hardware demands	Research applications, animal studies
pCASL	High SNR, clinically recommended by ISMRM	Sensitive to off-resonance effects	Routine perfusion imaging, cerebrovascular disorders
Multi-delay ASL	Estimates ATT, improves CBF quantification	Longer acquisition	Chronic cerebrovascular disease, CVR studies
VSASL	Sensitive to slow flow, ATT-independent	Lower SNR	White matter perfusion, microvascular disease
VE-ASL	Source-specific flow mapping	Longer acquisition, complex post-processing	Pre-surgical planning, vascular territory mapping

PASL, pulsed ASL; CASL, Continuous ASL; pCASL, pseudo-Continuous ASL; VSASL, Velocity selective ASL; VE-ASL, Vessel-encoded ASL; PLD, post labeling delay; RF, radiofrequency; CBF, cerebral blood flow; ATT, arterial transit time; CVR, cerebrovascular reactivity.

Despite its advantages, ASL perfusion MRI is highly sensitive to motion artifacts, susceptibility effects, partial volume errors, and low signal-to-noise ratio (SNR), all of which can hinder accurate CBF quantification. Motion artifacts cause geometric distortions and signal mismatches between the control and label images, and can be minimized using correction techniques, such as motion tracking or flow-triggered acquisitions, though patient cooperation remains essential (De Vis et al., 2014; Lu et al., 2012). Susceptibility artifacts, resulting from magnetic field inhomogeneities near tissue-air interfaces, can be reduced using techniques like VSASL or advanced post-processing algorithms such as Iterative Self-consistent Parallel Imaging Reconstruction (SPIRiT; Fan et al., 2021; Su et al., 2017; Inoue et al., 2014). Labeling efficiency and partial volume effects also impact CBF accuracy, with three-dimensional acquisitions helping to reduce these errors, albeit at the cost of longer scan times (McMorris et al., 2017; Hernandez-Garcia et al., 2022). Finally, the inherently low SNR of ASL, exacerbated by physiological and subtraction noise, can be improved through optimized acquisition protocols and digital filtering, ultimately enhancing measurement reliability (Grade et al., 2015).

### 2.2 Beyond CBF: ASL-derived physiological metrics

While CBF remains the core metric derived from ASL, other parameters accessible through tailored acquisition schemes or physiological challenges provide valuable insights into cerebrovascular health and adaptive capacity. These include arterial transit time (ATT), cerebrovascular reactivity (CVR), and cerebral metabolic rate of oxygen consumption (CMRO₂). These metrics expand the interpretive scope of ASL beyond perfusion alone, offering a more comprehensive view of brain hemodynamics and metabolism, particularly relevant in the context of hypoxic exposures (Table 2).

**Table 2:** Summary of derived metrics from ASL data.

Metric	Definition	Main application
Cerebral blood flow (CBF)	Volume of blood supplied to the brain per unit time (mL/100 g/min)	Assessing cerebral perfusion, important in hypoxia and pathology
Arterial transit time (ATT)	Time taken for labeled blood to reach the imaging region	Optimizing post-labeling delay (PLD), assessing cerebrovascular reactivity
Oxygen extraction fraction (OEF)	Fraction of oxygen extracted by brain tissue	Evaluating cerebral hypoxia, metabolic dysfunction, and tissue viability in stroke
Cerebral metabolic rate of oxygen (CMRO2)	The rate of oxygen consumption by the brain	Robust indicator of tissue viability in acute stroke and other conditions.
Cerebrovascular reactivity (CVR)	Cerebral vascular response to vasoactive challenges (e.g., hypercapnia, breath-holding)	Diagnosing chronic vascular diseases, predicting stroke risk

ATT is required for magnetically labeled blood to travel from the tagging plane to the tissue of interest. It reflects not only macrovascular transit but also microvascular flow resistance and collateral circulation status. ATT prolongation has been reported in conditions involving impaired vascular reserve, such as aging, cerebrovascular disease, and obstructive sleep apnea (Ishida, 2023; Woods et al., 2024; Li and Wang, 2023; Chen et al., 2012; Dai et al., 2017). Multi-delay ASL (MDASL) protocols are typically used to estimate ATT by acquiring signals at several PLDs, enabling robust modeling of the arrival time and separating it from true flow effects.

CVR measures the dynamic capacity of cerebral vessels to dilate or constrict in response to a vasoactive stimulus, such as CO₂ inhalation or breath-hold. ASL-based CVR mapping has been validated against BOLD-fMRI and PET techniques, and holds potential for detecting early dysfunction in neurovascular coupling. Reduced CVR may indicate compromised endothelial function, early vascular stiffness, or neuroinflammatory processes, particularly in chronic hypoxic conditions such as OSA or high-altitude exposure (Marshall et al., 2014; Solis-Barquero et al., 2021; Inoue et al., 2014). While CVR is not routinely measured in clinical ASL protocols, its incorporation is growing in advanced research settings due to its relevance in early diagnosis and intervention monitoring.

CMRO₂, the cerebral metabolic rate of oxygen consumption, provides a critical bridge between perfusion and metabolism. Although ASL does not directly measure CMRO₂, it can be estimated when ASL is combined with sequences that assess venous oxygenation, such as TRUST (T₂-Relaxation-Under-Spin-Tagging) or phase contrast MRI (Rodgers et al., 2015, 2016). These approaches exploit the Fick principle to estimate oxygen extraction fraction (OEF) and calculate CMRO₂ as the product of OEF and CBF. CMRO₂ mapping is particularly valuable in hypoxia, where perfusion increases may not always translate to metabolic sufficiency. For example, preserved or elevated CBF with reduced CMRO₂ may indicate luxury perfusion, while mismatched coupling could reflect tissue vulnerability or compensation failure (Jiang and Lu, 2022; De Vis et al., 2014; Lu et al., 2012; Hernandez-Garcia et al., 2022).

Taken together, these derived metrics position ASL as a multidimensional imaging technique capable of quantifying not only blood flow but also vascular timing, reactivity, and oxygen metabolism. This makes ASL particularly relevant for investigating hypoxia-induced neurovascular adaptations and dysfunctions across diverse contexts, from transient high-altitude exposure to chronic diseases like sleep apnea or cerebral small vessel disease.

## 3 ASL in environmental hypoxia

Hypoxia encountered in environmental contexts, such as high-altitude exposure or controlled experimental hypoxia, presents a valuable model for studying adaptive cerebrovascular responses in the absence of overt brain pathology. This section discusses the use of ASL to investigate acute and chronic forms of environmental hypoxia, emphasizing individual variability, metabolic compensation, and longitudinal vascular adaptation.

### 3.1 Acute environmental hypoxia

Acute exposure to hypoxia, whether in hypobaric chambers or sudden ascent to high altitudes, elicits rapid physiological adaptations in the brain to preserve oxygen delivery. ASL has been instrumental in revealing interindividual differences in these early responses, particularly through the mapping of CBF and derived indices, such as ATT and CVR.

In anoxic brain injury, ASL measurements have consistently reported globally enhanced CBF compared to controls. Notably, regions with increased diffusion signals also exhibit hyperperfusion, suggesting compensatory vasodilation in response to oxygen deprivation (Li et al., 2020). The loss of cerebrovascular autoregulation in these patients results in excessive CBF (mean gray matter CBF 142.6 mL/100 g/min), which may reflect a protective mechanism but also poses risks of edema and secondary injury (Pollock et al., 2008).

Across various forms of acute hypoxia, CBF increases are accompanied by alterations in the blood oxygenation index, suggesting a compensatory response to maintain oxygen delivery despite reduced oxygen availability (average SpO2 of 83.4%; Harris et al., 2013). This cerebrovascular response is particularly pronounced in regions involved in sensory processing, motor control, and cognitive functions, indicating that these areas are more sensitive to hypoxic stress and require greater perfusion to sustain their activity.

Similarly, ASL studies in high-altitude exposure show increased regional CBF, particularly in frontal and sensorimotor cortices, areas involved in cognition and motor control (Pagani et al., 2011; Buck et al., 1998). These adaptations reflect the brain’s attempt to maintain oxygen delivery despite reduced atmospheric pressure. Ventilatory and hematological compensations (e.g., hyperventilation, elevated hemoglobin levels) act synergistically to stabilize oxygen supply over time (Wang et al., 2018). In healthy subjects, this hyperperfusion correlates with elevated metabolic demand, reflected by increased CMRO₂ during initial hypoxic exposure (Dyer et al., 2008).

However, this balance is delicate. Studies indicate that CBF regulation may prioritize oxygen delivery over avoiding hyperperfusion, highlighting a flexible but imperfect adaptation strategy. For instance, under conflicting physiological demands, the brain may favor oxygen supply at the cost of higher perfusion pressure (Curtelin et al., 2018). This trade-off questions the reliability of using CBF alone as a proxy for cerebrovascular health during hypoxic stress.

Recent advances underscore the importance of integrating CBF, CVR, and CMRO₂ to capture the full spectrum of cerebrovascular adaptation. A multimodal MRI study by Deckers et al. (2022) combining ASL with venous oximetry showed that hypercapnia significantly increased CBF but reduced CMRO₂ when using CO₂ in air, challenging the assumption that such vasodilatory stimuli are metabolically inert. In contrast, carbogen (CO₂ + O₂) maintained CMRO₂ levels, suggesting that the addition of oxygen may buffer the metabolic consequences of vasodilation. These findings reveal that CVR and CBF changes can be decoupled from metabolic demand, and that measuring CMRO₂ is crucial for interpreting the physiological significance of perfusion alterations in hypoxia.

Furthermore, a recent systematic review emphasized that CVR is not a consistent predictor of CBF responses under hypoxia, pointing to distinct underlying mechanisms compared to hypercapnia-induced reactivity (Johnson et al., 2025). These insights challenge conventional interpretations of CVR and highlight the need for a multi-parametric approach to cerebrovascular assessment.

In contrast to hyperperfused states, mild hypoxia in pilots flying at high altitudes without supplemental oxygen presents a subtler yet concerning picture. Liu et al. (2021) reported decreased CBF in the right temporal, occipital, and cerebellar regions, areas critical for sensory integration, visuospatial processing, and motor coordination. This regional hypoperfusion, even before overt hypoxia symptoms, could impair reaction time, judgment, and flight safety. These findings raise operational concerns about subclinical cognitive deficits during prolonged exposure to mild hypoxia.

Taken together, these studies underscore the necessity of a dynamic, integrative framework, considering CBF, CVR, and CMRO₂, to understand environmental acute cerebrovascular responses to hypoxia. Such an approach provides a more physiologically grounded interpretation of ASL data and supports the development of robust biomarkers for monitoring cerebral resilience and vulnerability under environmental stress.

### 3.2 Chronic environmental hypoxia

Chronic exposure to environmental hypoxia, such as that experienced by high-altitude residents or through repeated hypoxic training such as in freediving (Micaux et al., 2025), induces long-term cerebrovascular adaptations that involve both vascular remodeling and metabolic reprogramming. Among the most consistently observed phenomena is the modulation of CBF and ATT, together providing a window into the evolving cerebrovascular status of the brain under sustained hypoxic stress.

Reductions in CBF are frequently reported during prolonged hypoxic exposure, especially in regions associated with the default mode network (DMN). Lawley et al. (2017) demonstrated that even short-term normobaric hypoxia (2–10 h) can lead to decreased perfusion in DMN hubs, an effect amplified over time and confirmed by hypercapnic challenges to be driven by vasoconstriction. This response may reflect a functional downregulation of resting-state activity under hypoxia, serving as an early indicator of cerebral adaptation. ATT, another crucial marker, may also be altered during chronic hypoxia. While some studies suggest a lengthening of ATT due to vascular rarefaction and reduced perfusion pressure, others report region-specific reductions in ATT driven by compensatory vasodilation and angiogenesis. The equation CBF = CBV / MTT (mean transit time), with ATT as a proxy for MTT in ASL protocols, illustrates how shifts in vessel diameter and volume dynamically impact cerebral hemodynamics. CO₂ accumulation, common in scenarios such as breath-holding, further modulates this relationship by acting as a potent vasodilator, simultaneously increasing cerebral blood volume and reducing ATT, thus amplifying CBF despite lowered oxygen availability (Keil et al., 2018).

Moreover, in chronic high-altitude conditions, inter-individual and ethnic variability in cerebrovascular responses becomes pronounced. For instance, Tibetans exhibit a 17% reduction in global CBF and a 22% increase in cerebrovascular resistance, in contrast to Han individuals who show more preserved CBF (Liu et al., 2016). Regionally, increased CBF has been observed in areas such as the inferior frontal gyrus and lentiform nucleus, possibly reflecting compensatory redistribution (Wang et al., 2018).

Additionally, animal models offer mechanistic insight into these adaptations. A murine study mimicking 5,000 m altitude exposure showed decreased hippocampal CBF, ventricular dilation, and white matter injury, alongside molecular evidence of neurovascular remodeling and myelin disruption (Cramer et al., 2019). Rats chronically exposed to 4,250 m similarly developed spatial memory impairments and hippocampal abnormalities that co-occurred with regional perfusion changes (Zhu et al., 2022).

Also, simulated microgravity studies using head-down tilt bed rest combined with elevated CO₂ (HDT + CO₂) have revealed differential CBF trajectories in individuals who developed spaceflight-associated neuro-ocular syndrome (SANS) compared to those who did not. While SANS participants showed an early drop in CBF followed by partial recovery, non-SANS individuals remained hypoperfused over 29 days, suggesting that chronic hypoxic and hypercapnic exposure unmask inter-individual differences in cerebrovascular regulation (Roberts et al., 2021).

These complex patterns underscore the inadequacy of considering CBF alone when evaluating cerebrovascular adaptation. Studies integrating hypercapnic reactivity and CMRO₂ measurements have shown that while some regions may exhibit increased perfusion, they do not always correspond to increased metabolic demand (Willie et al., 2014; Willie et al., 2015). Such dissociations highlight the importance of also quantifying ATT, CVR, and OEF to obtain a comprehensive understanding of neurovascular function.

**Figure 3:**
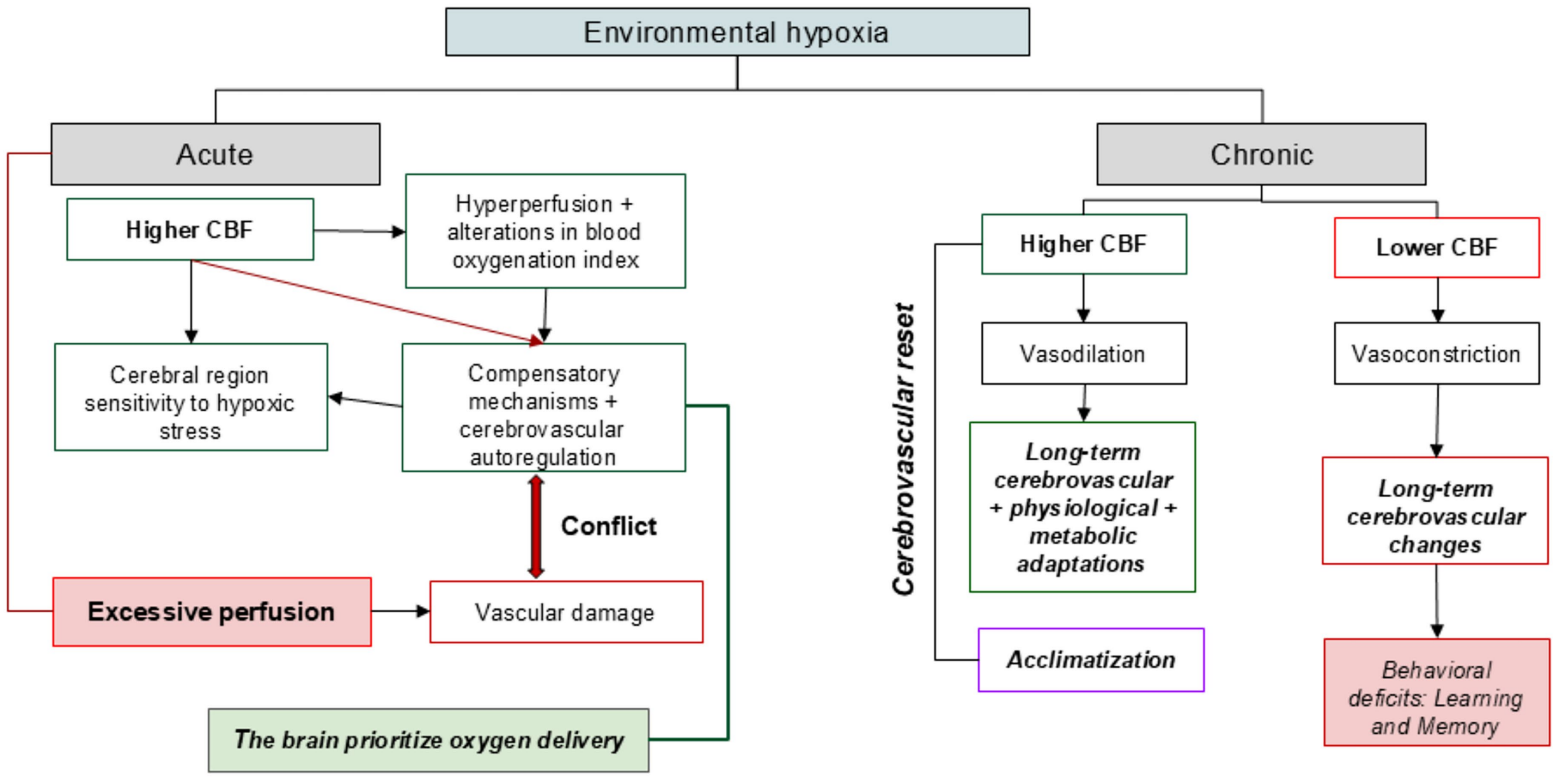
The brain’s vascular responses to environmental hypoxia under acute and chronic conditions. The color coding highlights the nature of each physiological process: Green: Positive adaptive responses or compensatory mechanisms that support cerebral oxygen delivery or neurovascular regulation (e.g., increased CBF, autoregulation). Red: Maladaptive responses or negative outcomes (e.g., excessive perfusion, vascular damage, behavioral deficits). Blue: Long-term adaptive changes involving cerebrovascular, physiological, and metabolic adjustments under chronic hypoxia. Purple: Acclimatization processes reflecting a specific type of long-term adaptation to hypoxic environments.

Ultimately, the chronic hypoxia-induced modulation of CBF and ATT offers critical insights into the brain’s capacity to recalibrate perfusion strategies over time. These parameters not only reflect the current state of vascular function but may also predict long-term consequences such as cognitive decline or structural degeneration. Their combined evaluation through multimodal neuroimaging approaches, incorporating ASL, venous oximetry, and diffusion tensor imaging, should be prioritized in future studies to better interpret the dynamic cerebrovascular responses and vulnerabilities associated with sustained environmental hypoxia (Figure 3; Table 2).

**Table:** 

Key insights: ASL in environmental hypoxia
Acute hypoxia triggers region-specific increases in CBF as a compensatory mechanism, particularly in sensory, motor, and cognitive regions.
This hyperperfusion may not always align with metabolic demand, emphasizing the need to jointly assess CBF, CVR, and CMRO₂.
Chronic environmental hypoxia leads to the region- and ethnicity-dependent cerebrovascular remodeling, involving both vascular (CBF, ATT) and metabolic (CMRO₂, OEF) adaptations.
Multimodal ASL studies reveal that traditional CVR markers may not fully capture the dynamic cerebrovascular responses to environmental hypoxic stress.

## 4 Hypoxia-induced pathological conditions

### 4.1 Acute hypoxia: pathophysiological insights from ASL

Acute hypoxia, as experienced in high-altitude exposure or perinatal asphyxia, elicits rapid cerebrovascular adaptations aiming to preserve oxygen delivery to metabolically active regions. This section explores the utility of ASL in two pathological contexts of acute hypoxia: high-altitude illness and HIE in neonates. It aims to determine (i) whether ASL-derived CBF metrics distinguish between vulnerable and resilient phenotypes, (ii) how CBF evolves dynamically with exposure duration, and (iii) whether ASL can capture compensatory or maladaptive responses contributing to neurological outcomes.

With increased human activity at high altitudes, AMS, and its severe form, high-altitude cerebral edema (HACE), have become prominent neurological syndromes (Lafuente et al., 2016). ASL studies have revealed potential predictive biomarkers of AMS, particularly through resting-state perfusion patterns at sea level. In a recent large-scale study, Zhang et al. (2024) demonstrated sex-specific predictors: men with higher cortical CBF, notably in the posterior circulation (e.g., right posterior cerebral artery), were more likely to develop AMS, while women exhibited a predictive asymmetry in anterior cerebral artery perfusion. These findings suggest that baseline CBF may reflect an individual’s vulnerability to AMS, although the mechanistic underpinnings remain unclear. Importantly, ASL also detects regional flow differences not captured by EEG or pulse oximetry, reinforcing its complementary diagnostic role (Feddersen et al., 2015). Also, AMS presents significant cerebrovascular challenges as demonstrated by Dyer et al. (2008). Despite no notable differences in oxygen saturation (SaO2) between AMS-susceptible (AMS-S) and AMS-resistant (AMS-R) individuals (87 ± 4% vs. 89 ± 3%, respectively), both groups exhibited increased whole-brain CBF during hypoxia.

Despite global increases in CBF during acute hypoxia, regional differences have not reliably distinguished AMS-S from resistant individuals. Liu et al. (2017) showed similar increases in both gray and white matter CBF between groups. However, magnetic resonance angiography indicated that individuals who developed AMS exhibited greater perfusion in large anterior vascular territories, suggesting that elevated flow in major arteries may relate to AMS symptoms, possibly through altered pressure gradients or venous outflow resistance. In parallel, CVR to CO₂ decreased significantly post-exposure, pointing to impaired vasodilatory capacity. These findings challenge earlier assumptions that linked AMS exclusively to hypoxia severity or oxygen saturation, instead highlighting the nuanced interplay between flow regulation and vascular reserve capacity.

The dynamics of CBF during and after high-altitude exposure reflect the transition from acute adaptation to long-term acclimatization. Initially, hypoxia-driven vasodilation predominates, leading to transient arterial enlargement despite hypocapnia-induced vasoconstriction. Over time, as ventilatory adaptation normalizes oxygenation, both CBF and artery diameters return toward sea-level baselines. Notably, Liu et al. (2023) reported a prolonged decline in regional CBF post-return, particularly in regions linked to neurocognitive symptoms. This decline correlated with elevated blood pressure, suggesting an interaction between systemic and cerebral hemodynamics (Liu et al., 2023). This post-hypoxic hypoperfusion raises concerns about long-term effects on brain function, emphasizing the need to monitor delayed cerebrovascular changes even after apparent acclimatization.

To better understand metabolic demand, Smith et al. (2013) assessed cerebral CMRO₂ and found increased values after 2 days at 3800 m, regardless of AMS status. This elevation may reflect heightened neural excitability due to reduced CO₂ from hyperventilation, but it does not appear to distinguish between affected and unaffected individuals. Consequently, while CMRO₂ increases are a general feature of acute hypoxia, they may not directly drive AMS development (Smith et al., 2013).

A critical yet understudied mechanism in AMS progression to HACE is venous compression. Using MRI over 22 h of high-altitude exposure, Sagoo et al. (2017) documented a progressive increase in white matter volume, correlated with AMS severity, and linked to restricted venous drainage in deep cerebral veins. This study underscores the contribution of venous outflow resistance to cerebral edema, a dimension not captured by arterial-focused metrics such as CBF alone (Sagoo et al., 2017).

ASL imaging reveals that high-altitude exposure triggers complex, regionally variable cerebrovascular responses. While elevated CBF is common, it does not reliably differentiate AMS risk. Instead, vulnerability appears linked to large-artery perfusion patterns, impaired CVR, and, potentially, venous drainage inefficiencies. The trajectory from hyperperfusion to post-exposure hypoperfusion, modulated by systemic factors such as blood pressure, suggests persistent brain vulnerability.

Furthermore, neonatal HIE represents another archetypal model of acute hypoxia with dynamic CBF changes caused by a lack of oxygen and/or blood flow to the brain around the time of birth. It represents a major cause of neonatal morbidity and mortality and is associated with long-term neurodevelopmental impairments (Greco et al., 2020).

ASL has revealed a characteristic evolution: an initial hypoperfusion phase (within 24 h), followed by compensatory hyperperfusion (days 1–3), and then region-specific persistence or normalization over the subacute period (days 4–28). These transitions reflect impaired autoregulation and vascular remodeling (Tang et al., 2019). The phase of hypoperfusion is followed by a period of compensatory hyperperfusion between days 1 and 3, where CBF rises markedly in most brain regions, excluding the frontal lobe, likely due to reactive vasodilation and blood flow redistribution toward metabolically vital deep structures (Tang et al., 2019; Wang et al., 2022; Meng et al., 2021). During the subsequent subacute stage (days 4 to 11), elevated CBF persists in selective areas, particularly the basal ganglia and temporal lobes, indicating ongoing vascular adaptation or delayed injury evolution (Proisy et al., 2019). From day 7 onward, and particularly between 15 to 28, studies report normalization of CBF across most brain regions, suggesting partial recovery of cerebral oxygenation and perfusion response to therapeutic interventions (Tang et al., 2019; Wang et al., 2022; Meng et al., 2021; Proisy et al., 2019).

Also, infants with severe HIE demonstrate the highest perfusion levels in the basal ganglia and thalamus, followed by those with moderate and mild injury. These deep regions are especially susceptible to hypoxic damage, and early CBF elevations, quantified using ASL, correlate with the degree of functional impairment, providing objective, visual indicators of injury severity and prognosis (Zheng et al., 2021b; Wang et al., 2021; Ji et al., 2023). Hypoperfusion in vital structures like the brainstem, cerebellum, and deep nuclei has been associated with poor neurodevelopmental scores (Bayley-III < 80), while hyperperfusion in these same regions correlates with more favorable outcomes (Zheng et al., 2020).

Beyond group-level findings, ASL holds strong prognostic potential. Studies report high diagnostic accuracy (AUC 0.97–0.99) when combining CBF metrics in deep structures with behavioral scores like NBNA (Liu et al., 2023). Moreover, ASL can detect perfusion changes even in HIE infants with normal conventional MRI, especially in regions linked to motor and language development (e.g., caudate, precentral gyrus; Cao et al., 2022).

Finally, sex-specific differences further refine interpretation: thalamic perfusion tends to be lower in male neonates, potentially contributing to higher vulnerability (Zheng et al., 2021a).

**Figure 4:**
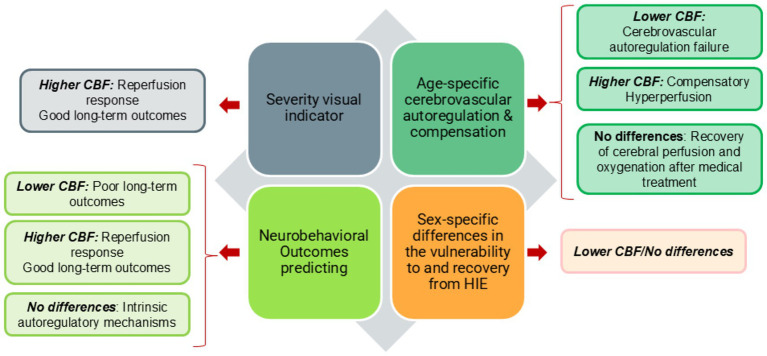
Cerebral blood flow (CBF) variations in hypoxic ischemic encephalopathy (HIE).

ASL provides a dynamic and sensitive biomarker of evolving brain perfusion in neonatal HIE. By characterizing the spatiotemporal trajectory of CBF, ASL helps distinguish between reversible versus progressive injury and predicts neurodevelopmental outcomes with high reliability (Figure 4).

#### 4.1.1 Chronic hypoxia in obstructive sleep apnea

OSA is characterized by recurrent upper airway obstruction during sleep, resulting in cycles of hypoxia and reoxygenation. These cycles can induce vascular oxidative stress, endothelial dysfunction, and impaired cerebral autoregulation (Claassen et al., 2021).

ASL studies have demonstrated both global and regional hypoperfusion in OSA patients, particularly those with moderate to severe disease (Chen et al., 2017). These reductions are frequently localized to functionally critical brain areas, including the corticospinal tracts, superior cerebellar peduncles, pontocerebellar fibers, thalamus, hippocampus, and insula, many of which are involved in motor control, arousal, and sensorimotor integration (Yadav et al., 2013). The asymmetrical nature of these impairments, such as lateralized hypoperfusion in the cerebellar peduncles, red nucleus, and midbrain, suggests localized vulnerability of motor coordination circuits, which may underlie the disordered synchrony between upper airway and diaphragmatic muscles characteristic of OSA.

Furthermore, perfusion deficits extend to cortical networks implicated in higher cognitive function. Decreased CBF has been observed in the DMN and central executive network (CEN), including the posterior cingulate cortex, dorsolateral parietal cortex, lateral prefrontal cortex, and inferior temporal lobes. These regions have been repeatedly linked to executive dysfunction, attention deficits, and memory impairments in OSA patients. Notably, CBF in memory-related areas such as the bilateral inferior temporal and left lingual gyri correlates positively with performance on delayed memory and attention tasks, suggesting a perfusion-based mechanism underlying cognitive decline (Nie et al., 2017; Yan et al., 2024). Moreover, hypoperfusion in deep gray matter structures, particularly the thalamus, caudate nucleus, and parahippocampal gyri, further implicates disrupted subcortical processing in the pathogenesis of neurobehavioral deficits (Yan et al., 2021).

Importantly, these perfusion abnormalities appear to be disease-severity dependent. While individuals with mild OSA often exhibit no significant differences in CBF compared to healthy controls, moderate to severe OSA is associated with extensive and consistent reductions in regional perfusion, particularly in areas already known to undergo structural degeneration (Innes et al., 2015). In some cases, such as the superior frontal gyrus, increased CBF has also been reported and shown to correlate with the longest apnea duration, possibly reflecting maladaptive or compensatory vasodilatory responses.

Compounding these changes, OSA patients demonstrate impaired CVR, especially in white matter, as evidenced by blunted CBF responses to hypercapnic challenges. This vascular dysregulation may compromise the brain’s ability to buffer ischemic stress and contribute to the elevated risk of cerebrovascular events such as stroke in this population (Ponsaing et al., 2018).

The neurovascular consequences of OSA extend further to blood–brain barrier dysfunction, which has been identified in patients with otherwise intact large vessel integrity (Palomares et al., 2015). These subtle but pervasive alterations in microvascular and barrier function likely promote chronic neuroinflammation and progressive neural injury (Postrzech-Adamczyk et al., 2019). Finally, OSA is also associated with a greater burden of white matter hyperintensities, further implicating microvascular compromise in its pathophysiology (Li et al., 2023).

**Figure 5:**
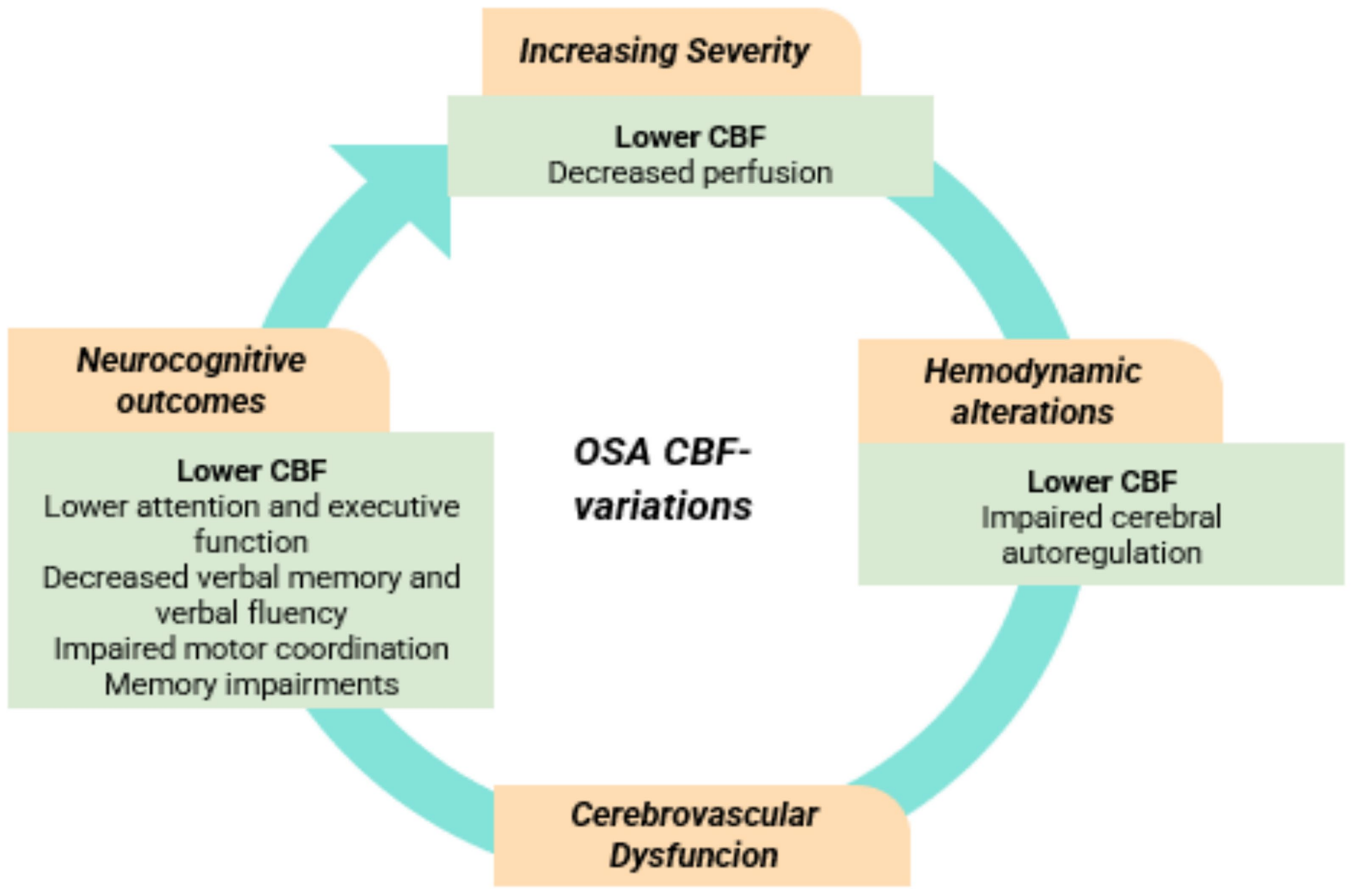
Cerebral blood flow (CBF) variations in obstructive sleep apnea (OSA) patients.

In conclusion, the evidence strongly supports a multifaceted impact of OSA on cerebral perfusion, affecting not only sensory-motor and arousal pathways but also higher-order cognitive networks. These perfusion abnormalities, often detectable even before overt structural brain damage, may serve as early neuroimaging biomarkers of neural compromise in OSA (Figure 5). The combination of reduced CBF, impaired CVR, and localized network dysfunction highlights a complex interplay between intermittent hypoxia, vascular dysregulation, and cognitive decline. Longitudinal and interventional studies are needed to determine the reversibility of these deficits and the extent to which therapies such as continuous positive airway pressure therapy can restore cerebral perfusion and preserve neurocognitive function (Buckley et al., 2024). Ultimately, integrating CBF imaging into clinical protocols may offer a valuable tool for the early detection, monitoring, and personalization of treatment in OSA.

**Table:** 

Key insights: ASL in hypoxia-induced pathological conditions
ASL identifies distinct perfusion patterns in pathological hypoxia (e.g., OSA, COPD, CHD), often revealing regional hypoperfusion linked to cognitive and vascular impairments.
In OSA, altered CBF and CVR patterns persist even during normoxic wakefulness, suggesting long-term neurovascular disruption.
Pediatric hypoxia shows both global and focal CBF alterations, with implications for neurodevelopment and plasticity.
Integrating ASL-derived CBF, ATT, and CVR with structural and functional data provides a sensitive approach to monitoring brain vulnerability in clinical hypoxia.

## 5 Discussion

### 5.1 Pathophysiological context of hypoxia: cerebrovascular adaptation

Hypoxia, whether acute or chronic, imposes profound challenges on the brain’s oxygen-dependent metabolism, triggering a cascade of cerebrovascular responses aimed at preserving neuronal viability. Due to its high metabolic demand and limited oxygen reserves, the brain is particularly susceptible to fluctuations in oxygen availability, from acute insults such as HIE or transient apneic events to sustained exposures like high-altitude residence or OSA (Umbrello et al., 2013). Understanding the balance between adaptive versus maladaptive vascular responses is critical for elucidating hypoxia-induced brain injury and optimizing preventive or therapeutic strategies.

In this context, ASL MRI has emerged as a pivotal neuroimaging tool. Its ability to non-invasively quantify CBF with high regional specificity, without requiring contrast agents, enables researchers and clinicians to monitor cerebrovascular function under hypoxic stress. This offers complementary or superior insights compared to conventional imaging modalities.

### 5.2 Acute hypoxia and perfusion dynamics

During acute hypoxia, ASL consistently reveals global or region-specific hyperperfusion, predominantly in metabolically demanding and oxygen-sensitive areas such as the prefrontal cortex, motor areas, and deep gray matter. This increased CBF reflects compensatory vasodilation aimed at preserving oxygen delivery despite reduced arterial oxygen content. Simultaneously, ATT often shortens, indicating accelerated blood flow and increased perfusion pressure. However, in severe hypoxia, autoregulatory capacity may be compromised, leading to maladaptive hyperperfusion and increased risk of oxidative stress or microvascular injury, particularly when CBF increases are uncoupled from CMRO₂, reflecting a mismatch between oxygen delivery and metabolic demand (Andjelkovic et al., 2020; Fajardo et al., 2022; Auer et al., 2008). These responses show regional and individual variability, influenced by vascular architecture, neurovascular coupling, and developmental or sex-specific factors. For example, in neonatal HIE, early hyperperfusion in the basal ganglia and thalamus has prognostic value for neurodevelopmental outcomes, with evidence of sex-based differences in perfusion vulnerability (Fajardo et al., 2022). These findings highlight the brain’s hierarchical strategy of preserving perfusion to essential functional networks during acute hypoxic episodes.

### 5.3 Chronic hypoxia and vascular remodeling

In contrast, chronic hypoxia, as seen in long-term high-altitude exposure or OSA, elicits more complex and heterogeneous vascular remodeling. While some regions maintain compensatory vasodilation, others, especially within the DMN and hippocampus, exhibit perfusion deficits likely reflecting metabolic downregulation and capillary rarefaction. Regionally specific ATT prolongation may indicate either vascular resistance or maladaptive remodeling processes such as microangiopathy or hypocapnia-induced vasoconstriction. These chronic adaptations are often accompanied by a persistent dissociation between CBF and CMRO₂, indicating impaired neurovascular and metabolic coupling (Inoue et al., 2014).

CVR, is frequently blunted in chronic hypoxia and, as detected by ASL, correlates with cognitive impairments in attention, executive function, and memory. Notably, inter-individual variability, as well as ethnic and developmental differences, modulate these responses, with potential implications for personalized risk stratification (Postrzech-Adamczyk et al., 2019).

### 5.4 Linking advanced ASL metrics to neuropsychological and functional outcomes

While advanced ASL approaches such as multi-delay protocols, VSASL, and vessel-encoded ASL provide detailed characterization of cerebral hemodynamics, relatively few studies have directly linked these parameters to comprehensive neuropsychological or functional assessments. Existing evidence suggests that alterations in CBF, ATT, CVR, and OEF are associated with domain-specific impairments, including reduced attention, executive dysfunction, and memory deficits in conditions such as obstructive sleep apnea, cerebrovascular disease, and chronic hypoxia (Johnson et al., 2025; Dai et al., 2017; Kim et al., 2023). However, the majority of these findings are correlative, with limited mechanistic exploration and sparse longitudinal follow-up. Integrating ASL-derived biomarkers with standardized cognitive batteries and functional measures could enable earlier detection of clinically meaningful changes, improve risk stratification, and guide targeted interventions. Future studies should prioritize multi-center, longitudinal designs that combine advanced ASL with robust neuropsychological profiling, thereby bridging the current gap between cerebral perfusion metrics and functional outcomes.

### 5.5 Gaps in longitudinal evidence and clinical implementation

**Figure 6:**
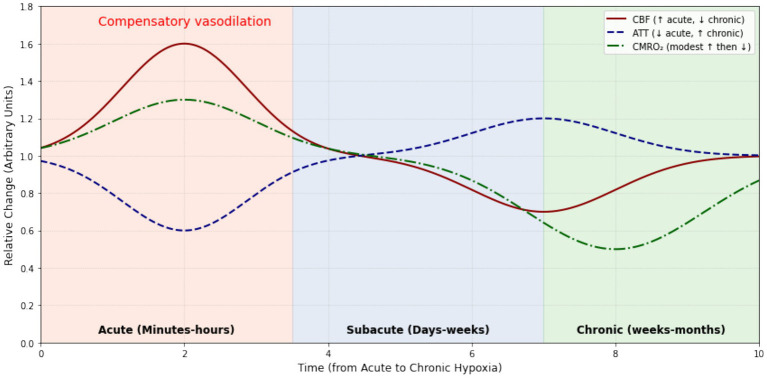
Conceptual evolution of CBF, ATT, and CMRO_2_ across hypoxia phases. CBF, cerebral blood flow; ATT, arterial transit time; CMRO_2_, cerebral metabolic rate of oxygen.

These distinct CBF patterns, hyperperfusion in acute hypoxia versus regional hypoperfusion and autoregulatory failure in chronic hypoxia, underscore the dynamic and phase-dependent nature of cerebral adaptation. ASL stands out for its ability to capture these spatiotemporal nuances, advancing our understanding of how the brain transitions from compensation to decompensation under prolonged oxygen stress (Figure 6; Table 3).

**Table 3:** Summary of cerebrovascular patterns in acute vs. chronic hypoxia across environmental and pathological contexts.

Dimension	Acute hypoxia	Chronic hypoxia	Context
Temporal onset	Minutes to hours (e.g., sudden altitude ascent, anoxic injury, apnea episodes)	Weeks–years (high-altitude residence, untreated OSA)	Environmental / pathological
CBF response	Global or regional hyperperfusion (sensory, motor, thalamus); compensatory	Variable or reduced CBF (DMN, hippocampus); downregulation, neurovascular uncoupling	ALL
ATT dynamics	Shortened (acute vasodilation and elevated perfusion pressure)	Prolonged or regionally altered (angiogenesis, rarefaction, CO₂ buffering)	ALL
CMRO2 coupling	Mismatch common: increased CBF > CMRO₂; risk of oxidative stress	Persistent decoupling in vulnerable regions (hippocampus, cortex)	Especially in OSA
CVR interpretation	May be unreliable (hypoxia ≠ hypercapnia reactivity)	Blunted or altered CVR (variable by ethnicity, disease severity)	Especially in chronic OSA and high-altitude
Functional impact	Transient deficits (reaction time, cognition)	Long-term cognitive decline (memory, executive function)	Pathological> environmental
Physiological mechanisms	No signaling, chemoreflex activation, ROS bursts	Angiogenesis, inflammation, vascular remodeling, blood barrier disruption	Environmental and pathological settings

Our review did not identify any longitudinal human studies directly linking early perfusion changes to long-term outcomes. Addressing this gap will require prospective cohort designs with repeated ASL measurements, integrated clinical follow-up, and incorporation of multimodal biomarkers. These efforts will be critical to defining the prognostic role of ASL in hypoxia-related brain disorders.

Moreover, the clinical implementation of ASL remains constrained by variability in acquisition protocols, including differences in labeling techniques, PLD settings, and post-processing pipelines, which complicates cross-study comparability. Furthermore, there is currently no universally accepted set of clinical guidelines defining when and how to incorporate ASL, particularly advanced techniques such as multi-delay ASL, into diagnostic workflows. While the ISMRM Perfusion Study Group consensus provides a valuable framework for standardized acquisition (Alsop et al., 2015), additional efforts are required to harmonize protocols across institutions and to validate reproducibility in multi-center settings. Recent works (Wang et al., 2023; Lindner et al., 2023; Paschoal et al., 2024) illustrate progress toward such standardization, yet widespread adoption will depend on establishing clear, evidence-based recommendations for clinical use.

### 5.6 Technical limitations, need for standardization and future perspectives

ASL remains sensitive to motion, low signal-to-noise ratio, partial volume effects, and susceptibility artifacts, challenges that are particularly relevant in pediatric, elderly, and clinically impaired populations. Advances such as velocity-selective ASL, multi-band imaging, improved background suppression, and machine learning–based motion and artifact correction are enhancing robustness and reproducibility (Hernandez-Garcia et al., 2022; Lindner et al., 2023). These developments are crucial for translating ASL into routine clinical practice while maintaining high data quality across diverse patient groups.

Emerging ASL techniques such as VSASL and multi-delay protocols provide enhanced sensitivity to OEF and microvascular dynamics, enriching our assessment of neurovascular coupling under hypoxic stress. Integrating ASL with BOLD-fMRI, diffusion imaging, and task-based paradigms can elucidate the functional consequences of perfusion alterations, from sensorimotor deficits to cognitive slowing (Post et al., 2024; Aguirre et al., 2005; Diekhoff et al., 2011; Suzuki et al., 2020; Poorman et al., 2020; Le et al., 2023). Furthermore, combining ASL with machine learning approaches applied to large, multimodal datasets may improve the early detection, classification, and monitoring of hypoxia-related brain pathologies (Hernandez-Garcia et al., 2022; Bhoi et al., 2024).

## 6 Conclusion

ASL perfusion MRI offers a powerful, non-invasive approach for characterizing the brain’s vascular responses to hypoxia. By enabling quantitative, region-specific measurement of CBF and related metrics, ASL provides critical insight into the distinction between adaptive and maladaptive cerebrovascular processes. Its utility spans experimental models, high-altitude studies, and pathological conditions such as HIE and OSA, bridging the gap between basic physiology and translational application. As this review highlights, acute hypoxia predominantly evokes a transient hyperperfusion, aiming to preserve cerebral oxygenation, whereas chronic hypoxia drives heterogeneous remodeling, often culminating in impaired autoregulation and neurovascular uncoupling. These differential patterns underscore the need for dynamic, multimodal imaging strategies. Future work should focus on refining multi-parametric ASL protocols, improving temporal resolution, and integrating computational modeling to more accurately map the trajectory of hypoxia-induced brain changes. Longitudinal studies combining ASL with metabolic, structural, and functional imaging will be instrumental in identifying early biomarkers, predicting cognitive outcomes, and guiding personalized therapeutic interventions for hypoxia-related brain disorders.

## References

[ref1] Aguirre G. K. Detre J. A. Wang J. (2005). Perfusion fMRI for functional neuroimaging. Int. Rev. Neurobiol. 66, 213–236. doi: 10.1016/S0074-7742(05)66007-2, PMID: 16387205

[ref2] Ainslie P. N. Subudhi A. W. (2014). Cerebral blood flow at high altitude. High Alt. Med. Biol. 15, 133–140. doi: 10.1089/ham.2013.1138, PMID: 24971767

[ref3] Alsop D. C. Detre J.-A. Golay X. Günther M. Hendrikse J. Hernandez-Garcia L.. (2015). Recommended implementation of arterial spin-labeled perfusion MRI for clinical applications: a consensus of the ISMRM perfusion study group and the European consortium for ASL in dementia. Magn. Reson. Med. 73, 102–116. doi: 10.1002/mrm.25197 24715426 PMC4190138

[ref4] Andjelkovic A. V. Stamatovic S. M. Phillips C. M. Martinez-Revollar G. Keep R. F. (2020). Modeling blood–brain barrier pathology in cerebrovascular disease in vitro: current and future paradigms. Fluids Barriers CNS 17:44. doi: 10.1186/s12987-020-00202-7, PMID: 32677965 PMC7367394

[ref5] Auer R. Dunn J. Sutherland G. (2008). Hypoxia and related conditions. Greenfield’s Neuropathol., 1, 63–119. doi: 10.1201/b13319-3

[ref6] Bhoi A. K. Panigrahi R. Victor H. C. Jhaveri R. H. (2024). Healthcare big data analytics: Computational optimization and cohesive approaches, vol. 10, Berlin/Boston: Walter de Gruyter GmbH & Co KG.

[ref7] Buck A. Schirlo C. Jasinsky V. Weber B. Burger C. von Schulthess G. K.. (1998). Changes of cerebral blood flow during short-term exposure to normobaric hypoxia. J. Cereb. Blood Flow Metab. 18, 906–910. 9701352 10.1097/00004647-199808000-00011

[ref8] Buckley R. J. Innes C. R. Kelly P. T. Hlavac M. C. Beckert L. Melzer T. R.. (2024). Cerebral perfusion is not impaired in persons with moderate obstructive sleep apnoea when awake. Sleep Breath. 28, 1609–1616. doi: 10.1007/s11325-024-03048-7, PMID: 38717716

[ref9] Cao J. Mu Y. Xu X. Li H. Liu Z. Cao M.. (2022). Cerebral perfusion changes of the basal ganglia and thalami in full-term neonates with hypoxic-ischaemic encephalopathy: a three-dimensional pseudo continuous arterial spin labelling perfusion magnetic resonance imaging study. Pediatr. Radiol. 52, 1559–1567. doi: 10.1007/s00247-022-05344-4, PMID: 35357515

[ref10] Chen H.-L. Lin H. C. Lu C. H. Chen P. C. Huang C. C. Chou K. H.. (2017). Systemic inflammation and alterations to cerebral blood flow in obstructive sleep apnea. J. Sleep Res. 26, 789–798. doi: 10.1111/jsr.12553, PMID: 28513057

[ref5006] Chen Y. Wang D. J. Detre J. A. (2012). Comparison of arterial transit times estimated using arterial spin labeling. MAGMA. 25, 135–144. doi: 10.1007/s10334-011-0276-5 21863374

[ref11] Claassen J. A. H. R. Thijssen D. H. J. Ronney B. Panerai R. B. Faraci F. M. (2021). Regulation of cerebral blood flow in humans: physiology and clinical implications of autoregulation. Physiol. Rev. 101, 1487–1559. doi: 10.1152/physrev.00022.2020, PMID: 33769101 PMC8576366

[ref12] Cramer N. P. Korotcov A. Bosomtwi A. Xu X. Holman D. R. Whiting K.. (2019). Neuronal and vascular deficits following chronic adaptation to high altitude. Exp. Neurol. 311, 293–304. doi: 10.1016/j.expneurol.2018.10.007, PMID: 30321497

[ref13] Curtelin D. Morales-Alamo D. Torres-Peralta R. Rasmussen P. Martin-Rincon M. Perez-Valera M.. (2018). Cerebral blood flow, frontal lobe oxygenation and intra-arterial blood pressure during sprint exercise in normoxia and severe acute hypoxia in humans. J. Cereb. Blood Flow Metab. 38, 136–150. doi: 10.1177/0271678X17691986, PMID: 28186430 PMC5757439

[ref14] Dai W. Fong T. Jones R. N. Marcantonio E. Schmitt E. Inouye S. K.. (2017). Effects of arterial transit delay on cerebral blood flow quantification using arterial spin labeling in an elderly cohort. J. Magn. Reson. Imaging 45, 472–481. doi: 10.1002/jmri.25367, PMID: 27384230 PMC5219871

[ref15] Dai W. Garcia D. De Bazelaire C. Alsop D. C. (2008). Continuous flow-driven inversion for arterial spin labeling using pulsed radio frequency and gradient fields. Magn. Reson. Med. 60, 1488–1497. doi: 10.1002/mrm.21790, PMID: 19025913 PMC2750002

[ref16] De Vis J. B. Petersen E. T. Alderliesten T. Groenendaal F. De Vries L. S. Van Bel F.. (2014). Non-invasive MRI measurements of venous oxygenation, oxygen extraction fraction and oxygen consumption in neonates. NeuroImage 95, 185–192. doi: 10.1016/j.neuroimage.2014.03.060, PMID: 24685437

[ref17] Deckers P. T. Bhogal A. A. Dijsselhof M. B. Faraco C. C. Liu P. Lu H.. (2022). Hemodynamic and metabolic changes during hypercapnia with normoxia and hyperoxia using pCASL and TRUST MRI in healthy adults. J. Cereb. Blood Flow Metab. 42, 861–875. doi: 10.1177/0271678X211064572, PMID: 34851757 PMC9014679

[ref18] Detre J. A. Alsop D. C. (1999). Perfusion magnetic resonance imaging with continuous arterial spin labeling: methods and clinical applications in the central nervous system. Eur. J. Radiol. 30, 115–124. 10401592 10.1016/s0720-048x(99)00050-9

[ref19] Detre J. A. Leigh J. S. Williams D. S. Koretsky A. P. (1992). Perfusion imaging. Magn. Reson. Med. 23, 37–45. 1734182 10.1002/mrm.1910230106

[ref20] Diekhoff S. Uludağ K. Sparing R. Tittgemeyer M. Cavuşoğlu M. von Cramon D. Y.. (2011). Functional localization in the human brain: gradient-echo, spin-echo, and arterial spin-labeling fMRI compared with neuronavigated TMS. Hum. Brain Mapp. 32, 341–357. doi: 10.1002/hbm.21024, PMID: 20533563 PMC6870385

[ref21] Dyer E. A. W. Hopkins S. R. Perthen J. E. Buxton R. B. Dubowitz D. J. (2008). Regional cerebral blood flow during acute hypoxia in individuals susceptible to acute mountain sickness. Respir. Physiol. Neurobiol. 160, 267–276. doi: 10.1016/j.resp.2007.10.010, PMID: 18088570 PMC2387187

[ref22] Fajardo A. Piper F. I. García-Cervigón A. I. (2022). The intraspecific relationship between wood density, vessel diameter and other traits across environmental gradients. Funct. Ecol. 36, 1585–1598. doi: 10.1111/1365-2435.14069

[ref23] Fan H. Su P. Huang J. Liu P. Lu H. (2021). Multi-band MR fingerprinting (MRF) ASL imaging using artificial-neural-network trained with high-fidelity experimental data. Magn. Reson. Med. 85, 1974–1985. doi: 10.1002/mrm.28560, PMID: 33107100

[ref24] Feddersen B. Neupane P. Thanbichler F. Hadolt I. Sattelmeyer V. Pfefferkorn T.. (2015). Regional differences in the cerebral blood flow velocity response to hypobaric hypoxia at high altitudes. J. Cereb. Blood Flow Metab. 35, 1846–1851. doi: 10.1038/jcbfm.2015.142, PMID: 26082017 PMC4635241

[ref25] Golay X. Hendrikse J. Lim T. C. C. (2004). Perfusion imaging using arterial spin labeling. Top. Magn. Reson. Imaging 15:10. doi: 10.1097/00002142-200402000-00003, PMID: 15057170

[ref26] Grade M. Hernandez Tamames J. A. Pizzini F. B. Achten E. Golay X. Smits M. (2015). A neuroradiologist’s guide to arterial spin labeling MRI in clinical practice. Neuroradiology 57, 1181–1202. doi: 10.1007/s00234-015-1571-z, PMID: 26351201 PMC4648972

[ref27] Greco P. Nencini G. Piva I. Scioscia M. Volta C. A. Spadaro S.. (2020). Pathophysiology of hypoxic–ischemic encephalopathy: a review of the past and a view on the future. Acta Neurol. Belg. 120, 277–288. doi: 10.1007/s13760-020-01308-3, PMID: 32112349

[ref28] Harris A. D. Murphy K. Diaz C. M. Saxena N. Hall J. E. Liu T. T.. (2013). Cerebral blood flow response to acute hypoxic hypoxia. NMR Biomed. 26, 1844–1852. doi: 10.1002/nbm.3026, PMID: 24123253 PMC4114548

[ref29] Hernandez-Garcia L. Aramendía-Vidaurreta V. Bolar D. S. Dai W. Fernández-Seara M. A. Guo J.. (2022). Recent technical developments in ASL: a review of the state of the art. Magn. Reson. Med. 88, 2021–2042. doi: 10.1002/mrm.29381, PMID: 35983963 PMC9420802

[ref30] Innes C. R. Kelly P. T. Hlavac M. Melzer T. R. Jones R. D. (2015). Decreased regional cerebral perfusion in moderate-severe obstructive sleep apnoea during wakefulness. Sleep 38, 699–706. doi: 10.5665/sleep.4658, PMID: 25669185 PMC4402676

[ref31] Inoue Y. Tanaka Y. Hata H. Hara T. (2014). Arterial spin-labeling evaluation of cerebrovascular reactivity to acetazolamide in healthy subjects. Am. J. Neuroradiol. 35, 1111–1116. doi: 10.3174/ajnr.A3815, PMID: 24371025 PMC7965130

[ref32] Ishida S. (2023). Estimation of cerebral blood flow and arterial transit time from multi-delay arterial spin labeling MRI using a simulation-based supervised deep neural network. J. Magn. Reson. Imaging 57, 1477–1489. doi: 10.1002/jmri.28433, PMID: 36169654

[ref33] Ji H. X. Tian Y. H. Hou W. S. Ying D. W. Deng K. X. (2023). The value of 3D arterial spin labeling in early diagnosis and short-term prognostic grouping of full-term neonatal hypoxic-ischemic encephalopathy. Res. Square. doi: 10.21203/rs.3.rs-2465094/v1

[ref34] Jiang D. Lu H. (2022). Cerebral oxygen extraction fraction MRI: techniques and applications. Magn. Reson. Med. 88, 575–600. doi: 10.1002/mrm.29272, PMID: 35510696 PMC9233013

[ref35] Johnson H. R. Wang M. C. Stickland R. C. Chen Y. Parrish T. B. Sorond F. A.. (2025). Variable cerebral blood flow responsiveness to acute hypoxic hypoxia. Front. Physiol. 16:1562582. doi: 10.3389/fphys.2025.1562582, PMID: 40144550 PMC11936916

[ref36] Keil V. C. Eichhorn L. Mutsaerts H. J. M. M. Träber F. Block W. Mädler B.. (2018). Cerebrovascular reactivity during prolonged breath-hold in experienced freedivers. AJNR Am. J. Neuroradiol. 39, 1839–1847. doi: 10.3174/ajnr.A5790, PMID: 30237299 PMC7410726

[ref5005] Kim D. Lipford M. E. He H. Ding Q. Ivanovic V. Lockhart S. N. (2023). Parametric cerebral blood flow and arterial transit time mapping using a 3D convolutional neural network. Magnetic Resonance in Medicine, 90, 583–595 37092852 10.1002/mrm.29674 PMC10847038

[ref37] Kwong K. K. Chesler D. A. Weisskoff R. M. Donahue K. M. Davis T. L. Ostergaard L.. (1995). MR perfusion studies with T1-weighted echo planar imaging. Magn. Reson. Med. 34, 878–887. 8598815 10.1002/mrm.1910340613

[ref38] Lafuente V. Bermudez G. Camargo-Arce L. Bulnes S. (2016). Blood-brain barrier changes in high altitude. CNS Neurolog. Disord. Drug Targets-CNS Neurolog. Disord. 15, 1188–1197. doi: 10.2174/1871527315666160920123911 27667557

[ref39] Lawley J. S. Macdonald J. H. Oliver S. J. Mullins P. G. (2017). Unexpected reductions in regional cerebral perfusion during prolonged hypoxia. J. Physiol. 595, 935–947. doi: 10.1113/JP272557, PMID: 27506309 PMC5285718

[ref40] Le L. N. Wheeler G. J. Holy E. N. Donnay C. A. Blockley N. P. Yee A. H.. (2023). Cortical oxygen extraction fraction using quantitative BOLD MRI and cerebral blood flow during vasodilation. Front. Physiol. 14:1231793. doi: 10.3389/fphys.2023.1231793, PMID: 37869717 PMC10588655

[ref41] Li X. Hui Y. Shi H. Li M. Zhao X. Li R.. (2023). Altered cerebral blood flow and white matter during wakeful rest in patients with obstructive sleep apnea: a population-based retrospective study. Br. J. Radiol. 96:20220867. doi: 10.1259/bjr.20220867, PMID: 36715135 PMC9975376

[ref42] Li Y. Wang Z. (2023). Deeply accelerated arterial spin labeling perfusion MRI for measuring cerebral blood flow and arterial transit time. IEEE J. Biomed. Health Inform. 27, 5937–5945. doi: 10.1109/JBHI.2023.3312662, PMID: 37812536 PMC10841663

[ref43] Li N. Wingfield M. A. Nickerson J. P. Pettersson D. R. Pollock J. M. (2020). Anoxic brain injury detection with the normalized diffusion to ASL perfusion ratio: implications for blood-brain barrier injury and permeability. AJNR Am. J. Neuroradiol. 41, 598–606. doi: 10.3174/ajnr.A6461, PMID: 32165356 PMC7144662

[ref44] Lindner T. Bolar D. S. Achten E. Barkhof F. Bastos‐Leite A. J. Detre J. A.. (2023). Current state and guidance on arterial spin labeling perfusion MRI in clinical neuroimaging. Magn. Reson. Med. 89, 2024–2047. doi: 10.1002/mrm.29572, PMID: 36695294 PMC10914350

[ref45] Liu J. Li S. Qian L. Xu X. Zhang Y. Cheng J.. (2021). Effects of acute mild hypoxia on cerebral blood flow in pilots. Neurol. Sci. 42, 673–680. doi: 10.1007/s10072-020-04567-3, PMID: 32654008

[ref46] Liu W. Liu J. Lou X. Zheng D. Wu B. Wang D. J.. (2017). A longitudinal study of cerebral blood flow under hypoxia at high altitude using 3D pseudo-continuous arterial spin labeling. Sci. Rep. 7:43246. doi: 10.1038/srep43246 28240265 PMC5327438

[ref47] Liu J. Liu Y. Ren L. H. Li L. Wang Z. Liu S. S.. (2016). Effects of race and sex on cerebral hemodynamics, oxygen delivery and blood flow distribution in response to high altitude. Sci. Rep. 6:30500. doi: 10.1038/srep30500 27503416 PMC4977556

[ref48] Liu Y. Yuan F. Peng Z. Zhan Y. Lin J. Zhang R.. (2023). Decrease in cerebral blood flow after reoxygenation is associated with neurological syndrome sequelae and blood pressure. Brain Sci. 13:1600. doi: 10.3390/brainsci13111600, PMID: 38002559 PMC10669967

[ref49] Lu H. Xu F. Grgac K. Liu P. Qin Q. Van Zijl P. (2012). Calibration and validation of TRUST MRI for the estimation of cerebral blood oxygenation. Magn. Reson. Med. 67, 42–49. doi: 10.1002/mrm.22970, PMID: 21590721 PMC3158970

[ref50] Mallat J. Rahman N. Hamed F. Hernandez G. Fischer M. O. (2022). Pathophysiology, mechanisms, and managements of tissue hypoxia. Anaesthesia Critical Care Pain Med. 41:101087. doi: 10.1016/j.accpm.2022.101087, PMID: 35462083

[ref5003] Marshall O. Lu H. Brisset J.-C. Xu F. Liu P. Brisset J.-C.. (2014). Impaired Cerebrovascular Reactivity in Multiple Sclerosis. JAMA Neurol. 71, 1275–1281. doi: 10.1001/jamaneurol.2014.1668 25133874 PMC4376108

[ref51] McMorris T. Hale B. J. Barwood M. Costello J. Corbett J. (2017). Effect of acute hypoxia on cognition: a systematic review and meta-regression analysis. Neurosci. Biobehav. Rev. 74, 225–232. doi: 10.1016/j.neubiorev.2017.01.019, PMID: 28111267

[ref52] Meng L. Wang Q. Li Y. Ma X. Li W. Wang Q. (2021). Diagnostic performance of arterial spin-labeled perfusion imaging and diffusion-weighted imaging in full-term neonatal hypoxic-ischemic encephalopathy. J. Integr. Neurosci. 20, 985–991. doi: 10.31083/j.jin2004099, PMID: 34997721

[ref53] Micaux J. Poiret C. Zhao J. El Hajj E. Tillenon M. Troudi Habibi A.. (2025). Does freediving lead to hippocampal adaptability to hypoxia and maintenance of episodic memory? J. Integr. Neurosci. 24:36672. doi: 10.31083/JIN36672 40767009

[ref54] Neumann K. Günther M. Düzel E. Schreiber S. (2022). Microvascular impairment in patients with cerebral small vessel disease assessed with arterial spin labeling magnetic resonance imaging: a pilot study. Front. Aging Neurosci. 14:871612. doi: 10.3389/fnagi.2022.871612, PMID: 35663571 PMC9161030

[ref55] Nie S. Peng D. C. Gong H. H. Li H. J. Chen L. T. Ye C. L. (2017). Resting cerebral blood flow alteration in severe obstructive sleep apnoea: an arterial spin labelling perfusion fMRI study. Sleep Breath. 21, 487–495. doi: 10.1007/s11325-017-1474-9, PMID: 28210922

[ref56] Noguchi T. Yoshiura T. Hiwatashi A. Togao O. Yamashita K. Nagao E.. (2008). Perfusion imaging of brain tumors using arterial spin-labeling: correlation with histopathologic vascular density. AJNR Am. J. Neuroradiol. 29, 688–693. doi: 10.3174/ajnr.A0903, PMID: 18184842 PMC7978189

[ref57] Pagani M. Salmaso D. Sidiras G. G. Jonsson C. Jacobsson H. Larsson S. A.. (2011). Impact of acute hypobaric hypoxia on blood flow distribution in brain. Acta Physiol. 202, 203–209. doi: 10.1111/j.1748-1716.2011.02264.x, PMID: 21323867

[ref58] Palomares J. A. Tummala S. Wang D. J. Park B. Woo M. A. Kang D. W.. (2015). Water exchange across the blood-brain barrier in obstructive sleep apnea: an MRI diffusion-weighted pseudo-continuous arterial spin labeling study. J. Neuroimaging 25, 900–905. doi: 10.1111/jon.12288, PMID: 26333175 PMC4562408

[ref59] Paschoal A. M. Woods J. G. Pinto J. Bron E. E. Petr J. Kennedy McConnell F. A.. (2024). Reproducibility of arterial spin labeling cerebral blood flow image processing: a report of the ISMRM open science initiative for perfusion imaging (OSIPI) and the ASL MRI challenge. Magn. Reson. Med. 92, 836–852. doi: 10.1002/mrm.30081, PMID: 38502108 PMC11497242

[ref60] Pollock J. M. Whitlow C. T. Deibler A. R. Tan H. Burdette J. H. Kraft R. A.. (2008). Anoxic injury-associated cerebral hyperperfusion identified with arterial spin-labeled MR imaging. Am. J. Neuroradiol. 29, 1302–1307. doi: 10.3174/ajnr.A1095, PMID: 18451089 PMC8119152

[ref61] Ponsaing L. B. Lindberg U. Rostrup E. Iversen H. K. Larsson H. B. Jennum P. (2018). Impaired cerebrovascular reactivity in obstructive sleep apnea: a case-control study. Sleep Med. 43, 7–13. doi: 10.1016/j.sleep.2017.10.010, PMID: 29482816

[ref62] Poorman M. E. Martin M. N. Ma D. McGivney D. F. Gulani V. Griswold M. A.. (2020). Magnetic resonance fingerprinting part 1: potential uses, current challenges, and recommendations. J. Magn. Reson. Imaging 51, 675–692. doi: 10.1002/jmri.26836, PMID: 31264748

[ref63] Post T. E. Denney C. Cohen A. Jordan J. Limper U. (2024). Human hypoxia models in aerospace medicine: potential applications for human pharmacological research. Br. J. Clin. Pharmacol. 1–15. doi: 10.1111/bcp.16040, PMID: 38556349 PMC12746364

[ref64] Postrzech-Adamczyk K. Nahorecki A. Łyszczak P. Skomro R. Szuba A. (2019). Impact of obstructive sleep apnea on cerebral blood flow. Postępy Hig. Med. Dośw. 73, 65–75. doi: 10.5604/01.3001.0013.0323

[ref65] Proisy M. Corouge I. Legouhy A. Nicolas A. Charon V. Mazille N.. (2019). Changes in brain perfusion in successive arterial spin labeling MRI scans in neonates with hypoxic-ischemic encephalopathy. NeuroImage: Clin. 24:101939. doi: 10.1016/j.nicl.2019.101939, PMID: 31362150 PMC6664197

[ref66] Qin Q. Alsop D. C. Bolar D. S. Hernandez-Garcia L. Meakin J. Liu D.. (2022). Velocity-selective arterial spin labeling perfusion MRI: a review of the state of the art and recommendations for clinical implementation. Magn. Reson. Med. 88, 1528–1547. doi: 10.1002/mrm.29371, PMID: 35819184 PMC9543181

[ref5002] Rodgers Z. B. Detre J. A. Wehrli F. W. (2016). MRI-based methods for quantification of the cerebral metabolic rate of oxygen. Journal of Cerebral Blood Flow & Metabolism, 36, 1165–1185. 27089912 10.1177/0271678X16643090 PMC4929705

[ref5001] Rodgers Z. B. Englund E. K. Langham M. C. Magland J. F. Wehrli F. W. (2015). Rapid T2-and susceptometry-based CMRO2 quantification with interleaved TRUST (iTRUST). Neuroimage, 106, 441–450. 25449740 10.1016/j.neuroimage.2014.10.061 PMC4576991

[ref67] Roberts D. R. Collins H. R. Lee J. K. Taylor J. A. Turner M. Zaharchuk G.. (2021). Altered cerebral perfusion in response to chronic mild hypercapnia and head-down tilt bed rest as an analog for spaceflight. Neuroradiology 63, 1271–1281. doi: 10.1007/s00234-021-02660-8, PMID: 33587162

[ref68] Sagoo R. S. Hutchinson C. E. Wright A. Handford C. Parsons H. Sherwood V.. (2017). Magnetic resonance investigation into the mechanisms involved in the development of high-altitude cerebral edema. J. Cereb. Blood Flow Metab. 37, 319–331. doi: 10.1177/0271678X15625350, PMID: 26746867 PMC5167111

[ref69] Smith Z. M. Krizay E. Guo J. Shin D. D. Scadeng M. Dubowitz D. J. (2013). Sustained high-altitude hypoxia increases cerebral oxygen metabolism. J. Appl. Physiol. 114, 11–18. doi: 10.1152/japplphysiol.00703.2012, PMID: 23019310 PMC3544513

[ref5004] Solis-Barquero S. M. Echeverria-Chasco R. Calvo-Imirizaldu M. Cacho-Asenjo E. Martinez-Simon A. Vidorreta M.. (2021). Breath-hold induced cerebrovascular reactivity measurements using optimized Pseudocontinuous arterial spin labeling. Frontiers in Physiology. 12:621720. doi: 10.3389/fphys.2021.621720 33679436 PMC7925895

[ref70] Su P. Mao D. Liu P. Li Y. Pinho M. C. Welch B. G.. (2017). Multiparametric estimation of brain hemodynamics with MR fingerprinting ASL. Magn. Reson. Med. 78, 1812–1823. doi: 10.1002/mrm.26587, PMID: 28019021 PMC5484761

[ref71] Suzuki Y. Clement P. Dai W. Dolui S. Fernández-Seara M. A. Lindner T.. (2024). ASL lexicon and reporting recommendations: a consensus report from the ISMRM Open Science initiative for perfusion imaging (OSIPI). Magn. Reson. Med. 91, 1743–1760. doi: 10.1002/mrm.29815, PMID: 37876299 PMC10950547

[ref72] Suzuki Y. Fujima N. van Osch M. J. P. (2020). Intracranial 3D and 4D MR angiography using arterial spin labeling: technical considerations. Magn. Reson. Med. Sci. 19, 294–309. doi: 10.2463/mrms.rev.2019-0096, PMID: 31761840 PMC7809141

[ref73] Tang S. Liu X. He L. Liu B. Qin B. Feng C. (2019). Application of a 3D pseudocontinuous arterial spin-labeled perfusion MRI scan combined with a postlabeling delay value in the diagnosis of neonatal hypoxic-ischemic encephalopathy. PLoS One 14:e0219284. doi: 10.1371/journal.pone.0219284, PMID: 31283776 PMC6613698

[ref74] Taso M. Alsop D. C. (2024). Arterial spin labeling perfusion imaging. Magn. Reson. Imaging Clin. N. Am. 32, 63–72. doi: 10.1016/j.mric.2023.08.005, PMID: 38007283

[ref75] Troudi A. Tensaouti F. Baudou E. Péran P. Laprie A. (2022). Arterial spin labeling perfusion in pediatric brain tumors: a review of techniques, quality control, and quantification. Cancer 14:4734. doi: 10.3390/cancers14194734, PMID: 36230655 PMC9564035

[ref76] Troudi A. Tensaouti F. Cabarrou B. Arribarat G. Pollidoro L. Péran P.. (2023). A prospective study of arterial spin labelling in paediatric posterior fossa tumour survivors: a correlation with neurocognitive impairment. Clin. Oncol. 36, 56–64. doi: 10.1016/j.clon.2023.09.015 37805352

[ref77] Umbrello M. Dyson A. Feelisch M. Singer M. (2013). The key role of nitric oxide in hypoxia: hypoxic vasodilation and energy supply–demand matching. Antioxid. Redox Signal. 19, 1690–1710. doi: 10.1089/ars.2012.4979, PMID: 23311950

[ref78] Wang J. Alsop D. C. Li L. Listerud J. Gonzalez-At J. B. Schnall M. D.. (2002). Comparison of quantitative perfusion imaging using arterial spin labeling at 1.5 and 4.0 tesla. Magn. Reson. Med. 48, 242–254. doi: 10.1002/mrm.10211, PMID: 12210932

[ref79] Wang X. Bishop C. O'Callaghan J. Gayhoor A. Albani J. Theriault W.. (2023). MRI assessment of cerebral perfusion in clinical trials. Drug Discov. Today 28:103506. doi: 10.1016/j.drudis.2023.103506, PMID: 36690177

[ref80] Wang J. Jia L. Xiaoping Y. Huan Z. Zheng Y. Huaijun L. (2022). Cerebral hemodynamics of hypoxic-ischemic encephalopathy neonates at different ages detected by arterial spin labeling imaging. Clin. Hemorheol. Microcirc. 81, 271–279. doi: 10.3233/CH-211324, PMID: 35253735

[ref81] Wang J. Li J. Yin X. Zhou H. Zheng Y. Ma X.. (2021). The value of arterial spin labeling imaging in the classification and prognostic evaluation of neonatal hypoxic-ischemic encephalopathy. Curr. Neurovasc. Res. 18, 307–313. doi: 10.2174/1567202618666210920112001, PMID: 34544339

[ref82] Wang X. Wei W. Yuan F. Li S. Lin J. Zhang J. (2018). Regional cerebral blood flow in natives at high altitude: an arterial spin labeled MRI study. J. Magn. Reson. Imaging 48, 708–717. doi: 10.1002/jmri.25996, PMID: 29493838

[ref83] Williams D. S. Detre J. A. Leigh J. S. Koretsky A. P. (1992). Magnetic resonance imaging of perfusion using spin inversion of arterial water. Proc. Natl. Acad. Sci. 89, 212–216. 1729691 10.1073/pnas.89.1.212 PMC48206

[ref84] Willie C. K. MacLeod D. B. Smith K. J. Lewis N. C. Foster G. E. Ikeda K.. (2015). The contribution of arterial blood gases in cerebral blood flow regulation and fuel utilization in man at high altitude. J. Cereb. Blood Flow Metab. 35, 873–881. doi: 10.1038/jcbfm.2015.4, PMID: 25690474 PMC4420871

[ref85] Willie C. K. Smith K. J. Day T. A. Ray L. A. Lewis N. C. S. Bakker A.. (2014). Regional cerebral blood flow in humans at high altitude: gradual ascent and 2 wk at 5,050 m. J. Appl. Physiol. 116, 905–910. doi: 10.1152/japplphysiol.00594.2013, PMID: 23813533

[ref86] Wong E. C. (2007). Vessel-encoded arterial spin-labeling using pseudocontinuous tagging. Magn. Reson. Med. 58, 1086–1091. doi: 10.1002/mrm.21293, PMID: 17969084

[ref87] Wong W. H. E. Maller J. J. (2016). Arterial spin labeling techniques 2009–2014. J. Med. Imag. Radiation Sci. 47, 98–107. doi: 10.1016/j.jmir.2015.08.002, PMID: 31047171

[ref88] Woods J. G. Achten E. Asllani I. Bolar D. S. Dai W. Detre J. A.. (2024). Recommendations for quantitative cerebral perfusion MRI using multi-timepoint arterial spin labeling: acquisition, quantification, and clinical applications. Magn. Reson. Med. 92, 469–495. doi: 10.1002/mrm.30091, PMID: 38594906 PMC11142882

[ref89] Yadav S. K. Kumar R. Macey P. M. Richardson H. L. Wang D. J. J. Woo M. A.. (2013). Regional cerebral blood flow alterations in obstructive sleep apnea. Neurosci. Lett. 555, 159–164. doi: 10.1016/j.neulet.2013.09.033, PMID: 24076138 PMC3891908

[ref90] Yan X. Liu W. Li D. Huang Q. Wu J. Zhang Q. (2024). Decreased memory-related regional cerebral perfusion in severe obstructive sleep apnea with a mild cognitive impairment during wakefulness. Nature Sci. Sleep 16, 1869–1880. doi: 10.2147/NSS.S481602, PMID: 39649801 PMC11624665

[ref91] Yan L. Park H. R. Kezirian E. J. Yook S. Kim J. H. Joo E. Y.. (2021). Altered regional cerebral blood flow in obstructive sleep apnea is associated with sleep fragmentation and oxygen desaturation. J. Cereb. Blood Flow Metab. 41, 2712–2724. doi: 10.1177/0271678X211012109, PMID: 33906511 PMC8504950

[ref92] Zhang H. Feng J. Zhang S. Y. Liu W. J. Ma L. (2024). Predicting Acute Mountain sickness using Regional Sea-level cerebral blood flow. Biomed. Environ. Sci. 37, 887–896. doi: 10.3967/bes2024.100, PMID: 39198253

[ref93] Zheng Q. Freeman C. W. Hwang M. (2021a). Sex-related differences in arterial spin-labelled perfusion of metabolically active brain structures in neonatal hypoxic–ischaemic encephalopathy. Clin. Radiol. 76, 342–347. doi: 10.1016/j.crad.2020.12.026, PMID: 33579516 PMC8935139

[ref94] Zheng Q. Martin-Saavedra J. S. Saade-Lemus S. Vossough A. Zuccoli G. Gonçalves F. G.. (2020). Cerebral pulsed arterial spin labeling perfusion weighted imaging predicts language and motor outcomes in neonatal hypoxic-ischemic encephalopathy. Front. Pediatr. 8. doi: 10.3389/fped.2020.576489, PMID: 33102411 PMC7546822

[ref95] Zheng Q. Viaene A. N. Freeman C. W. Hwang M. (2021b). Radiologic-pathologic evidence of brain injury: hypoperfusion in the Papez circuit results in poor neurodevelopmental outcomes in neonatal hypoxic ischemic encephalopathy. Childs Nerv. Syst. 37, 63–68. doi: 10.1007/s00381-020-04795-0, PMID: 32661642 PMC7796967

[ref96] Zhu D. He B. Zhang M. Wan Y. Liu R. Wang L.. (2022). A multimodal MR imaging study of the effect of hippocampal damage on affective and cognitive functions in a rat model of chronic exposure to a plateau environment. Neurochem. Res. 47, 979–1000. doi: 10.1007/s11064-021-03498-5, PMID: 34981302 PMC8891211

